# *In Vitro* Differentiation of Neural Stem Cells into Noradrenergic-Like Cells

**Published:** 2015

**Authors:** Vahid Pirhajati Mahabadi, Mansoureh Movahedin, Saeed Semnanian, Javad Mirnajafi-zadeh, Mehrdad Faizi

**Affiliations:** 1 *Anatomical Sciences Department, Faculty of Medicine, Tarbiat Modares University, Tehran, Iran.*; 2 *Physiology Department, Faculty of Medicine, Tarbiat Modares University, Tehran, Iran.*; 3 *Pharmacology and Toxicology Department, Faculty of Pharmacy, Shahid Beheshti University of Medical Sciences, Tehran, Iran.*

**Keywords:** Neural stem cells, sub- ventricular zone, noradrenergic- like cells

## Abstract

Neural stem cells (NSCs) as a heterogeneous multipotent and self- renewal population are found in different areas in the developing mammalian nervous system, as well as the sub-ventricular zone (SVZ) and the hippocampus of the adult brain. NSCs can give rise to neurons, astrocytes and oligodendrocytes. The aim of this study was to differentiate neural stem cells into noradrenergic–like cells *in vitro*. Neural stem cells were harvested from SVZ of newborn rat brains. The cells were cultured in DMEM12, B-27 supplemented with 20 ng/ ml (hFGF) and 20 ng/ ml (EGF) for 2 weeks. Neurospheres were differentiated in neurobasal medium, B-27 supplemented with BDNF (50 ng/ ml) and GDNF (30 ng/ ml) for 3 and 5 days. Cell culture techniques and immunocytochemistry were applied to examine neurospheres and tyrosine hydroxylase positive cells. The number of neurites was counted 3 and 5 days after the induction of differentiation. Nestin and Sox2 were expressed in NSCs and neurospheres. NSCs were differentiated into noradrenergic- like cells (NACs). Tyrosine hydroxylase was detected in these cells. The results of NSCs differentiation for 5 days culture had a significant decrease (P≤0.05) in the number of TH positive cells with one or two neurite per cell, and a significant increase (P≤0.05) in the number of TH positive cells with three, four or more neurites per cell, compared with 3 days culture. Based on these results, NSCs have the ability to differentiate into noradrenergic cells in the presence of BDNF and GDNF growth factors.

Neural Stem Cell (NSC) is a multipotent cell which is able to self- renew and proliferate. NSC resides in a variety of areas in the developing mammalian nervous system as well as in the sub- ventricular zone (SVZ) and the hippocampus of the adult brain (1, 2). These cells can generate multiple neural lineages, including neurons, astrocytes and oligodendrocytes (3, 4).

The SVZ is a region in the brain that is situated throughout the lateral walls of the lateral ventricles. It is a known site of neurogenesis and self- renewing neurons in the adult brain, serving as such due to the interacting cell types, extracellular molecules and localized epigenetic regulation promoting such cellular proliferation (5). Recent *in vitro* studies of NSC based neurogenesis and gliogenesis have proposed that these processes occur by stepwise limitation and are dependent on environmental signals. Control of NSC proliferation related to the actions of epidermal growth factor (EGF) and/ or its homolog transforming growth factor, basic fibroblast growth factor (FGF-2), may form free floating aggregates termed neurospheres (6-8). NSC derived neur0000osphere, switch to asymmetric division cycles and give rise to another stem cell and one progenitor cell. The progenitor cells just have the potential to develop into other progenitor cells. Anyway, each clonal neurosphere consists of only a small amount of real stem cells (9). GDNF, RET (receptor tyrosine kinase molecule) and the GDNF co- receptor GFRα1 are expressed by central noradrenergic neurons in regions of the pons, including the A5 and A6 (nucleus locus coeruleus) cell groups (10). GDNF and BDNF are important for the survival, maintenance and regeneration of specific neuronal populations in the adult brain (11, 12). GDNF enhances catecholaminergic differentiation of various neuronal cell types in culture (13, 14). A5 development culture analysis shows that GDNF affect during early fetal developmental stage to promote the differentiation of noradrenergic neurons without displaying a survival or proliferation change (15). However, GDNF cannot act individually and requires a cofactor like brain-derived neurotrophic factor (BDNF) (15).

Previous studies showed that NSCs can be differentiated into neurons and glial cells (3, 4). But so far no study has been done on the differentiation of these cells into NACs. Although, an intrinsic neurogenesis and gliogenesis self- repair takes place endogenously during adulthood, NSCs are unable to reconstitute and restore function fully after extensive damage in adult brains. So, the aim of the present study was to evaluate the *in vitro* differentiation of NSCs into noradrenergic- like cells as a source of adult stem cell for treatment of neurological diseases in future.

## Materials and methods

**Culture of NSCs**

Sub-ventricular zones were harvested aseptically from 5 Wistar newborn rat brains. This work was developed under the approval of the Ethics Committee of Tarbiat Modarres University.

The brains were sectioned under magnifying lens in a petri dish containing PBS (phosphate buffered saline). SVZs were dissociated from lateral ventricles then the tissues were digested for 5min at 37 °C in 0.02% trypsin (Invitrogen, UK) plus 0.002% deoxyribonuclease I (Sigma, Germany). The tissues were gently dissociated with a Pasteur pipette into single cells, then plated and cultured in Dulbecco’s Modified Eagle Medium F12 (DMEM /F12) (Gibco, USA) with 2% B-27 serum- free supplement (Gibco, USA), 2 mM L- glutamine (Gibco, USA), 1% penicillin (Invitrogen, UK), and 1% streptomycin (Invitrogen, UK) for 24h. Then the cells were transferred to non- coated 6- well plates (PFL, Korea) containing the above medium. For initial culture, 20ng/ ml hFGF (Royan, Iran), 20 ng/ ml hEGF (Calbiochem, USA) were added and the cells were grown for 2 weeks) (3). One half of the culture medium was replaced with fresh medium twice per week. During culture, cells grew as clusters which eventually lost contact with the flask surface and grew as spherical structures (‘‘neurospheres’’). In all of these media, dissociated single cells divided and formed neurospheres by day 14. Neurospheres were dissociated with a Pasteur pipette into single cells. Cells were passaged by mechanical dissociation after 14 days and reseeded at approximately 1× 10^5^ cells/ cm^2^. Cells viability was assessed using Trypan Blue (Sigma, Germany)(3).

**NSCs differentiation into noradrenergic- like cells**

NSCs were dissociated into single cells. NSCs and human neuron- committed teratocarcinoma (NT2) cell line (as positive control) were plated onto Poly- D- lysine (100 g/ ml; Millipore, USA) and Laminin (0.3 g/ ml; Sigma, Germany) coated 12- well plates (PFL, Korea) in Neurobasal medium (Gibco, USA) supplemented with B- 27 serum- free supplement (Gibco, USA), 0.5 mM L- glutamine (Gibco, USA), at a density of 3.4×10^4^ cells per cm^2^. Trophic factors BDNF (50 ng/ ml; Sigma, UK) and GDNF (30 ng/ ml, Peprotech, UK) were added to the wells for 3 and 5 days (15).

**Identification of neurospheres and NACs**

For phenotype characterization, neurospheres or dissociated cells were plated on coverslips coated with gelatin in DMEM /F12 with 2% B- 27 serum- free supplement, 2 mM L- glutamine, hFGF and hEGF for 3 days. To evaluate NSCs differentiation into neurons and glial cells, dissociated cells from neurospheres were cultured in DMEM F12 without hFGF and hEGF for 14 days, then were plated on coverslips coated with gelatin in above culture medium for 2 days. For differentiation into Noradrenergic- like cells, NSCs and NT2 cells (positive control) were cultured in differentiation medium (GDNF, BDNF) for 3 and 5 days. Then the cells were fixed for 20 min at room temperature with 4% paraformaldehyde (Sigma, Germany) and washed three times in PBS. The cultures then were incubated in 2N HCl for 30 min at room temperature and washed with borate buffer for 15min, and incubated with blocking solution containing 10% goat serum (Invitrogen, UK) and 0.3% Triton X-100 (Sigma, Germany) in PBS for 30min at room temperature and incubated with primary antibodies overnight at 4 °C. The primary antibodies used were polyclonal anti-human Sox 2 antibody (anti- Sox 2) (Abcam, UK), polyclonal anti- Nestin antibody (Abcam, UK), polyclonal anti- glial fibrillary acid protein (anti- GFAP) (Abcam, UK), polyclonal anti- NG2 antibody, chondroitin sulfate proteoglycan (anti-NG2) (Abcam, UK) and monoclonal anti- neuron specific class III beta tubulin antibody (anti- b III tubulin) (Abcam, UK). The cells were then washed with PBS and incubated for 2h at room temperature in the dark with polyclonal secondary antibody FITC- conjugated goat anti- rabbit IgG (Abcam, UK). The cells subsequently were washed three times in PBS, and cell nuclei were counterstained with PI (3). Also, noradrenergic- like cells and NT2 cells were incubated overnight at 4 °C with polyclonal primary antibody anti- TH (Tyrosine hydroxylase) (Abcam, UK). The following day, primary antibody was rinsed with PBS and cells were incubated for 2h at room temperature with polyclonal secondary antibody FITC- conjugated goat anti -rabbit IgG (Abcam, UK). Then, the cells were washed with PBS and cover slipped (15). The specimens were examined by fluorescent microscope (AX70TRF, Olympus, Japan) and randomly picked fields of sections (30 individual fields of three independent samples) were captured, using a magnification of 20× objective lens. OLYSIA Bio Report Software was used for counts of primary neuritis. Quantification was estimated by the percentage of positive cells (expressed specific marker) in comparison to the total cells, which stained with PI in the fields.

**Statistical Analysis**

Data have been presented as the mean± SD with at least three biological independent repeats. Independent sample T- test was used to analyze group differences of the resultant data. The difference between groups was considered as statistically reliable if p≤0.05.

## Results

**Expansion and characterization of NSCs**

Dissociated cells were grown in serum- free medium containing hFGF and hEGF for 2 weeks to stimulate proliferation. In all cultures, The cells proliferated in the form of clusters or neurospheres. When the clusters grew larger, they detached from the bottom of flasks and became suspended in the medium, forming neurospheres. Most neurospheres were spherical. All cells were disaggregated mechanically after 14 days. Neurospheres formed after proliferation in DMEM/ F12 medium with hEGF and hFGF. To characterize these cells, we performed immunocytochemistry with the antibodies against Sox 2 and Nestin as specific markers for NSCs.

**Fig. 1:**
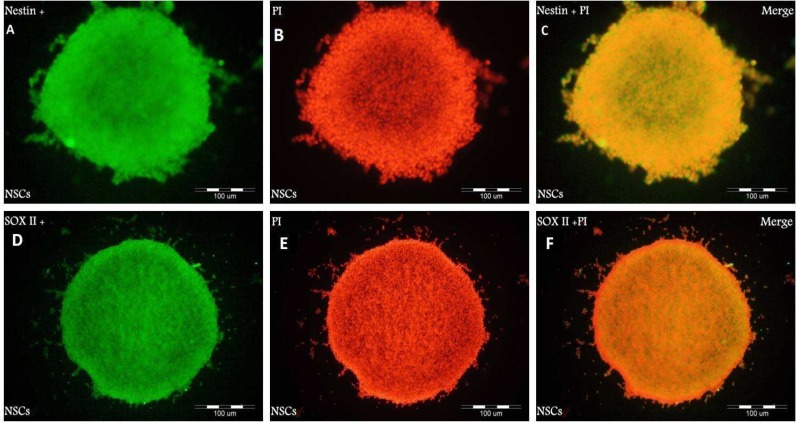
Immunocytochemical analysis of neurosphere with the antibodies against Nestin (green: A) and Sox- 2 (green: D) markers for neural stem cells. PI was used to stain nuclei (red: B, E) and Merged images (C, F).

**Fig. 2:**
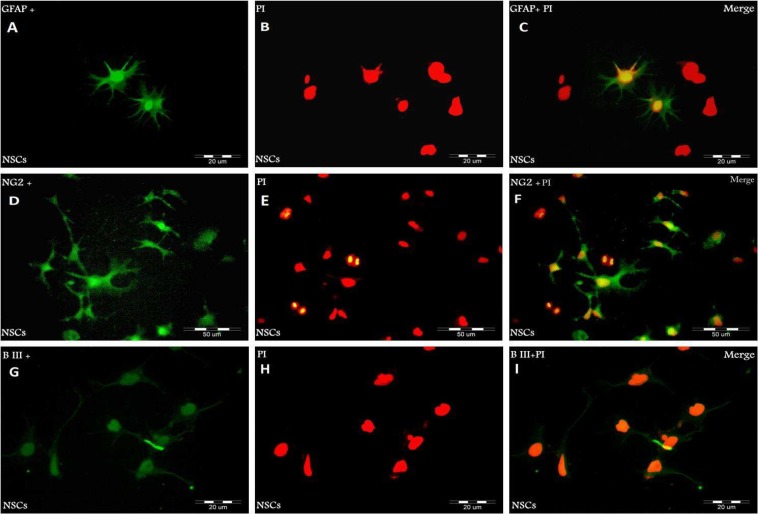
Immunocytochemical analysis of differentiated NSCs with antibodies against GFAP (green: A), NG2 (green: D) and b- III tubulin (green: G). The time to induce differentiation was 14 days. PI was used to stain nuclei (red:B, E, H) and Merged images (C, F, I).

As shown in Fig. 1 A– F, neurospheres were Nestin and Sox 2 positive, suggesting that they are NSCs. We examined whether NSCs had the potential to differentiate into both glial and neuronal cell types. Neurospheres were dissociated into single cells and induced to differentiation in DMEM/ F12 medium containing 5% serum without growth factors (hFGF, hEGF) for 14 days. GFAP (a marker for astrocytes), NG2 (a marker for oligodendrocyte precursor cells) and lineage- specific markers, b-III tubulin (a marker for neurons), were used to characterize NSCs after the induction of differentiation. 14 days after induction of differentiation, NSCs differentiated into either GFAP- positive astrocytes, NG2- positive oligodendrocyte lineage cells or b-III tubulin- positive neurons (Fig. 2 A– I).

**Effect of growth factors on undifferentiated NSCs proliferation**

This study demonstrates that in the medium containing hFGF and hEGF, NSCs remain undifferentiated and proliferate extensively. The combination of hEGF and hFGF in the medium enhanced the proliferation of NSCs. Also, the elimination of hEGF and hFGF from medium can differentiate these cells into neurons, astrocytes and oligodendrocytes (Fig. 2 A– I).

**Effect of GDNF and BDNF on differentiation of NSCs**

**Fig. 3:**
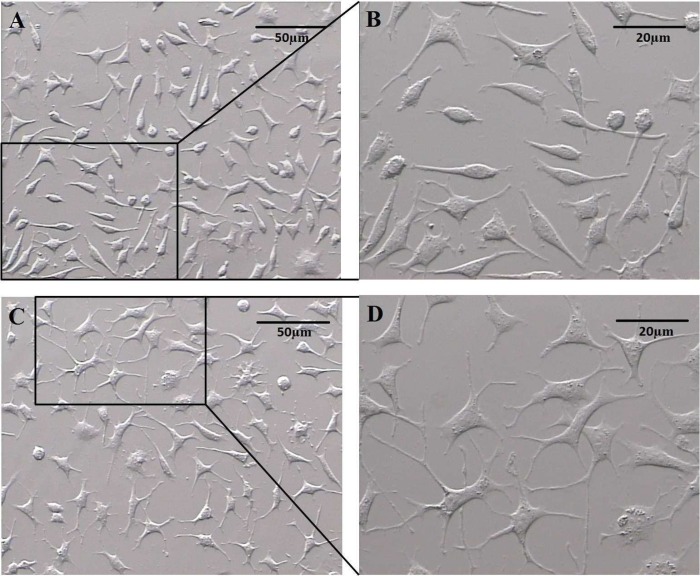
Phase contrast images of noradrenergic- like cells. the primary neurites arising from the cell body after 3 (A, B) and 5 (C, D) days culture in differentiation medium

**Fig. 4:**
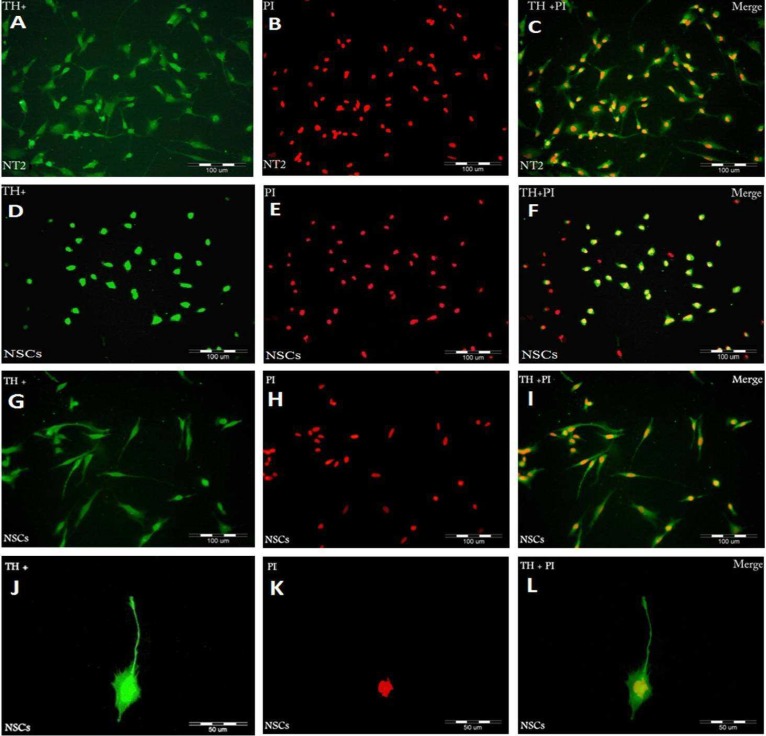
Immunocytochemical analysis of Tyrosine hydroxylase (TH) positive cells with antibodies against TH. NT2 (Green: A) and NSCs (Green: D, G, J). The time to induce differentiation in A, D was 3 days and G, J was 5 days. PI was used to stain nuclei (Red: B, E, H, K) and Merged images (C, F, I, L).

NSCs were cultured in differentiation medium for 3 and 5 days. We examined whether GDNF and BDNF regulate morphologic differentiation of NSCs by comparing the number of primary neurites expressed by TH positive cells. Fig. 3 shows phase contrast images of the primary neurites arising from the cell body of noradrenergic- like cells after 3 and 5 days culture in differentiation medium. After 3 days culture, in NT2 cell line (positive control), most TH positive cells were spindle shaped or polygonal with three primary neurites per cell (Fig. 4 A- C), whereas in NSCs, most TH positive cells were round or spindle shaped and exhibited one or two primary neurites arising from the cell body (Fig. 4 D- F), but after 5 days NSCs culture, most TH positive cells exhibited three, four or more primary neurites per cell (Fig. 4 G- L).

**Fig. 5:**
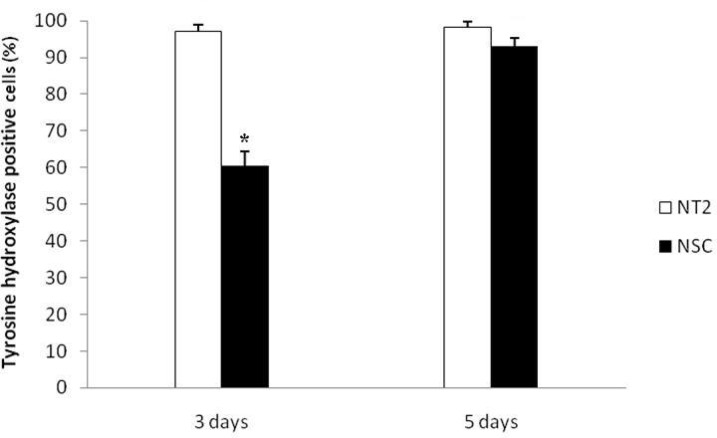
The results of NT2 cells (positive control) and NSCs differentiation into Tyrosine Hydroxylase (TH ) positive cells after 3 and 5 days culture .* Significant differences with other groups (P≤0.05).

**Fig. 6:**
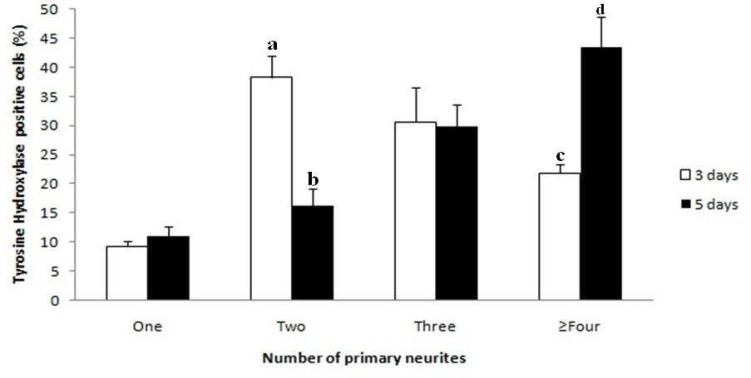
Effect of GDNF and BDNF on the number of primary neurites after 3 and 5 days culture. In each category, there are significant differences between groups with different alphabets.

There was a significant difference in the number of TH positive cells for NSCs differentiation after 5 days (93± 2.23) in comparison to 3 days culture (60.6± 3.78) (Fig. 5). Counts of primary neurites after 5 days culture indicate that there is a significant decrease (P≤ 0.05) in the number of TH positive cells with one or two primary neurites per cell, and a significant increase (P≤ 0.05) in the number of TH positive cells with three, four or more neurites per cell, compared with 3 days culture (Fig. 6). However, these data indicate that GDNF and BDNF are required for the differentiation of NSCs into noradrenergic- like cells *in vitro*.

## Discussion

Neurogenesis occurs mostly in two germinal regions of the adult mammalian brain: the SVZs line the most of the lateral walls of the lateral ventricles (16) and the subgranular layer of the dentate gyrus in the hyppocampus (17). These zones are the largest germinal zones of the adult mammalian brain. Proliferation of SVZ cells have been identified in several vertebrate species including human, mouse, rats, rabbits, dogs and cows (18-20).

In this study, we isolated NSCs from SVZs of newborn rat brains. NSCs were expanded as neurospheres in serum-free suspension cultures supplemented with hEGF and hFGF growth factors and these neurosphere expressed Sox 2 and Nestin markers. In Doetsch et al.'s study human neural progenitor cells (hNPCs) were allowed to reform into neurospheres. These cells were positive for both GFAP and b-III tubulin at 10 days post differentiation (21, 22). In contrast with Doetsch et al., in 2007, Wang et al. showed that feline NPCs (fNPCs) might not have fully differentiated at 10 days of induction. This study showed that differentiated cells were positive for either GFAP or b-III tubulin after 25 days post induction (3).

Previous studies have confirmed that hEGF and hFGF2 are essential growth factors for proliferation of hNPCs (3, 23). The results of our study revealed that NSCs differentiated into b-III tubulin positive neurons, GFAP positive astrocytes and NG2 positive oligodendrocyte precursor cells after 14 days.

Wang et al. found that using a combination of growth factors such as hEGF and hFGF can increase neurospheres formation from NSCs (3). Moreover, adding human leukemia inhibitory factor (hLIF) can promote long term proliferation of hFGF2- or hEGF-responsive hNPCs (24).

NSCs differentiated into neuron and glial cells in response to differentiation medium supplemented with 5% serum without hEGF and hFGF and also formed neurospheres in the presence of hEGF and hFGF (3).

GDNF also plays an essential role for the development of noradrenergic neurons in the pontine A5 cell group *in vivo* and *in vitro* (15). It enhances noradrenergic differentiation of neurons in the pontine A5 noradrenergic cells and BDNF acts as a cofactor to apply this effect. It is shown clearly that the number of TH positive A5 neurons significantly decrease in GDNF null mutant mice. Therefore, GDNF is required for the development of this population of noradrenergic neurons *in vivo* (15).

In this study, TH positive cells within each culture were evaluated according to the number of neurites arising from the cell body. The results showed that GDNF and BDNF increased the number of neurites *in vitro*.

The present study demonstrates that the addition of both GDNF and BDNF is able to induce differentiation of NSCs into noradrenergic- like cells and similar to other studies (15) enhance neurites formation in these cells. Furthermore, the percentage of TH positive cells after 5 days culture had a significant increase in comparison to 3 days culture. It has been observed that GDNF has similar effects in the differentiation of mesencephalic dopamine neurons (25, 26). Both GDNF and BDNF are simultaneously necessary to production of dopaminergic primary sensory neurons *in vivo* (27).

Our study identified that NSCs from SVZs can expand as neurospheres in serum- free suspension cultures supplemented with hEGF and hFGF growth factors. Neurospheres were positive for Sox 2 and Nestin markers. Also, we showed that GDNF and BDNF are required for the differentiation of NSCs into noradrenergic- like cells *in vitro* and increased the number of neurites after 5 days culture. These findings can be useful for research in neurological diseases field due to the damage to the pons and other brain regions containing noradrenergic cells.
